# Characteristics, Comorbidities, and Data Gaps for Coronavirus Disease Deaths, Tennessee, USA

**DOI:** 10.3201/eid2710.211070

**Published:** 2021-10

**Authors:** John James Parker, Rany Octaria, Miranda D. Smith, Samantha J. Chao, Mary Beth Davis, Celia Goodson, Jon Warkentin, Denise Werner, Mary-Margaret A. Fill

**Affiliations:** Tennessee Department of Health, Nashville, Tennessee, USA (J.J. Parker, R. Octaria, M.D. Smith, S.J. Chao, M.B. Davis, C. Goodson, J. Warkentin, D. Werner, M.-M.A. Fill);; Vanderbilt University Medical Center, Nashville (J.J. Parker)

**Keywords:** coronavirus disease, COVID-19, severe acute respiratory syndrome coronavirus 2, SARS-CoV-2, coronaviruses, viruses, respiratory infections, ethnic groups, comorbidities, underlying conditions, mortality rates, epidemiology, population characteristics, public health surveillance, Black patients, White patients, Hispanic patients, zoonoses, Tennessee, United States

## Abstract

As of March 2021, coronavirus disease (COVID-19) had led to >500,000 deaths in the United States, and the state of Tennessee had the fifth highest number of cases per capita. We reviewed the Tennessee Department of Health COVID-19 surveillance and chart-abstraction data during March 15‒August 15, 2020. Patients who died from COVID-19 were more likely to be older, male, and Black and to have underlying conditions (hereafter comorbidities) than case-patients who survived. We found 30.4% of surviving case-patients and 20.3% of deceased patients had no comorbidity information recorded. Chart-abstraction captured a higher proportion of deceased case-patients with >1 comorbidity (96.3%) compared with standard surveillance deaths (79.0%). Chart-abstraction detected higher rates of each comorbidity except for diabetes, which had similar rates among standard surveillance and chart-abstraction. Investing in public health data collection infrastructure will be beneficial for the COVID-19 pandemic and future disease outbreaks.

As of March 5, 2021, the total of deaths from coronavirus disease (COVID-19) reached 2,564,560 worldwide, 515,013 in the United States (*1*), and 11,534 in Tennessee (*2*). Tennessee has been particularly affected by the pandemic; as of March 5, 2021, this state had the fifth highest number of cases per 100,000 residents in the United States (*3*). The mortality rate for COVID-19 infection varies greatly based on patient characteristics (*4*,*5*). Age and preexisting health conditions (hereafter comorbidities) have been associated with increased risk for death from COVID-19 (*5**–**7*). Cardiovascular disease (CVD), hypertension, diabetes, respiratory disease, cancer, kidney disease, and obesity have been associated with death; however, the strength of this association has differed among studies (*5**,**7*). Although worldwide racial and ethnic minorities account for a higher proportion of COVID-19 deaths, the independent impact of race and ethnicity is unclear (*8*).

Challenges with data collection and reporting have made it difficult to delineate some characteristics of COVID-19 deaths. According to an assessment of surveillance data reported to the Centers for Disease Control and Prevention (CDC), 58.9% of patients had missing comorbidity information (*6*). Because public health agencies gather their surveillance information from local laboratories and healthcare facilities; the completeness of their data are contingent on the local agencies obtaining and transmitting the information (*9*,*10*). Consequently, mortality rate studies often focus on medical record reviews from single institutions and urban centers (*11*–*13*).

To better distinguish the characteristics of COVID-19 deaths, during March 15, 2020‒May 19, 2020, the Tennessee Department of Health (TDH) implemented a supplemental chart-abstraction process for COVID-19 deaths in Tennessee. This study reviews TDH COVID-19 surveillance data and the supplemental chart review data to describe the characteristics of COVID-19 deaths in Tennessee. In addition, this study evaluates the value of a supplemental chart review process during disease outbreak surveillance.

## Methods

Our study describes TDH public health data that was collected as part of COVID-19 surveillance. Definitions and protocols in place were defined by the TDH, who used CDC guidelines for their definitions of confirmed cases, probable cases, and COVID-19 deaths (*14*). Confirmed case-patients in Tennessee were defined as persons who had SARS-CoV-2 detected by using real-time reverse transcription PCR. Probable case-patients were persons who had a positive antigen test result for a respiratory specimen or persons who had no positive PCR result but met the vital records criteria or clinical criteria and had close contact to a COVID-19 case-patient during the 14 days before illness onset (*15*). COVID-19 deaths were defined as case-patients whose death certificate lists COVID-19 or SARS-CoV-2 as an underlying cause of death or a major condition contributing to death (*16*).

The sample included confirmed and probable cases in Tennessee residents who had COVID-19. We conducted investigations during March 15, 2020‒August 15, 2020. Data analysis began on September 15; we used a minimum 4-week lag time to best ensure that case-patients were categorized as alive or deceased. Our primary objective was to evaluate the baseline characteristics and comorbidities of persons who died from COVID-19 in Tennessee. A secondary objective was to compare the type and quantity of data obtained through standard disease surveillance and a supplemental chart review process. The TDH Institutional Review Board (TDH-IRB# 2020–0251) approved this study as minimal risk and waived the need for individualized consent.

### Data Collection

As part of routine data entry for all COVID-19 cases, trained TDH employees completed the Human Infection with 2019 Novel Coronavirus Case Report Form (CRF) (https://www.cdc.gov/coronavirus/2019-ncov/downloads/pui-form.pdf) (*17*) and entered the information into the National Electronic Disease Surveillance System Base System (NBS; https://www.cdc.gov/nbs/index.html). Information gathered included patient characteristics, symptoms, comorbidities, and clinical course. (The terms preexisting condition and comorbidity were used by TDH to indicate medical conditions that were present before COVID-19 infection; these terms are used interchangeably in this article.) Data collected through the CRF was the TDH standard COVID-19 disease surveillance.

In addition, during the first few months of the pandemic, the TDH created a supplemental chart review process to better classify the comorbidities and characteristics of deceased patients. This chart-abstraction project began with a group of public health professionals creating a list of 20 comorbidities to supplement the information in the standard CRF (Appendix 1). The chart review process creates line items for additional comorbidities. However, the CRF has 2 free text items for other chronic diseases and other underlying conditions. Therefore, we believe there is value in comparing comorbidity frequencies between the data collection groups.

Next, we added the additional chart-abstraction comorbidities to NBS to enable data entry. We then requested the medical records of all COVID-19 patients who died before May 19, 2020; 5 physicians and 1 family medicine nurse practitioner reviewed the available medical records. The provider group only reviewed complete records that included at least a complete history and physical or complete death summary. This provider group abstracted the information from the charts and added comorbidities found in the medical records to the NBS database. If there was no mention of a comorbidity, we assumed that the person did not have an underlying condition. However, when charts had gaps in documentation, the medical providers included comorbidities if there was clear evidence that the patient had a condition. For example, if a patient’s chart had minimal medical history documented but had chronic problems listed in a note’s plans, those problems were recorded as preexisting conditions. The group met and decided on definitions of diseases, and if there was any question on how a disease should be categorized, the individual provider would consult the group. For haste of getting this information to public health leadership, the provider’s chart-abstraction work was not reviewed by a second party. After completing their review, the providers updated the information from the CRF and added additional comorbidity data into NBS. Data from the supplemental chart review project were labeled as chart-abstracted. Preliminary data from the chart review project were presented to TDH leadership at the end of May 2020.

### Data Characterization and Analysis

**Figure 1:**
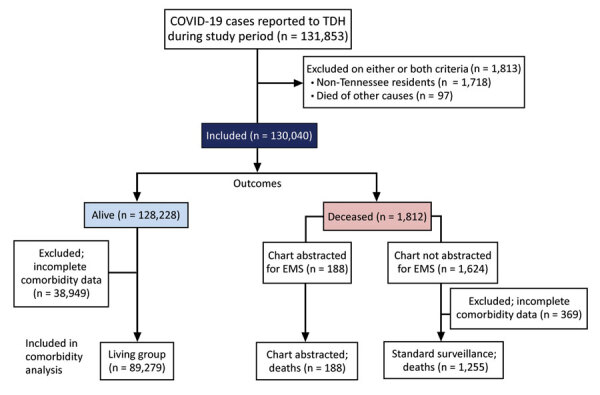
Data categorization flow diagram for characteristics, comorbidities, and data gaps for coronavirus disease deaths, Tennessee, USA. COVID-19, coronavirus disease; EMS, emergency medical services; TDH, Tennessee Department of Health.

We grouped COVID-19 case-patients into 3 groups: alive (living) case-patients, standard surveillance COVID-19 deaths, and chart-abstracted COVID-19 deaths (Figure 1). All COVID-19 cases (n = 130,040) during the study period were included in demographic analysis (Table 1). The comorbidity analysis (Tables 2, 3; Appendix 2) excluded case-patients who had no comorbidity information recorded by only selecting cases with >1 answers completed in the comorbidity or preexisting condition sections (n = 89,270). In both the standard surveillance and the chart-abstraction process, if comorbidity data in the CRF was partially completed, blank items were listed as not having that condition. For race/ethnicity, we defined White as White race, non-Hispanic ethnicity, and Black as Black race, non-Hispanic ethnicity. We defined Hispanic as all races that selected Hispanic ethnicity. For our race/ethnicity comorbidity analysis (Tables 2, 3; Appendix 2), we excluded all other races because there were only 6 case-patients in the chart-abstracted group who were not identified as White, Black, or Hispanic.

**Table 1:** Characteristics of coronavirus disease patients in Tennessee, USA, March 15–August 15, 2020

Characteristic	Living, n = 128,228	Deceased, n = 1,812
Race and ethnicity, no. (%)		
White, non-Hispanic	56,271 (43.9)	1,093 (60.3)
Black, non-Hispanic	26,697 (20.8)	540 (29.8)
Hispanic (all races)	21,390 (16.7)	126 (7.0)
All other races	8,155 (6.4)	49 (2.7)
Unknown	15,715 (12.3)	4 (0.2)
Sex, no. (%)		
F	64,966 (50.7)	789 (43.5)
M	62,100 (48.4)	1,023 (56.5)
Unknown	1,162 (0.9)	0
Age group, y, no. (%)		
0–20	21,703 (16.9)	4 (0.2)
21–64	93,486 (72.9)	451 (24.9)
65−80	10,080 (7.9)	706 (39.0)
>81	2,820 (2.2)	651 (35.9)
Unknown	139 (0.1)	0
Age group, y, has any comorbidity, no. (%)*		
21–64†	20,844 (22.3)	252 (55.9)
65–80	5,043 (50.0)	467 (66.2)
>81	1441 (51.1)	454(69.7)
All age groups	28,925 (22.6)	1,174 (64.8)
Incomplete comorbidity data, no. (%)‡	38,949 (30.4)	369 (20.3)
Mean (SD) days to hospitalization§	3.9 (5.4)	3.2 (5.8)
Mean (SD) days for obtaining specimen	1.8 (3.7)	1.8 (4.1)

*Includes persons who have incomplete comorbidity; data counted as having no comorbidity. †Cell percent is the percentage within that age group (i.e., for living case-patients 21–64 years of age, the percentage is 20,844/94,486). ‡Incomplete comorbidity data were defined as any chart without a single recorded response for any preexisting condition or comorbidity. §Days to hospitalization for living patients are only calculated for case-patients hospitalized because of coronavirus disease (n = 5,379).

**Table 2:** Case-fatality rate stratified by race and age for characteristics, comorbidities, and data gaps for coronavirus disease deaths, Tennessee, USA

Characteristic	White, non-Hispanic, n = 46,677	Black, non-Hispanic, n = 16,669	Hispanic, n = 17,084
Mean age, y (interquartile range)	40 (25‒57)	37 (25‒52)	32 (21‒45)
Age group, y, case-fatality rate, no. (%)*	896 (1.9)	428 (2.6)	84 (0.5)
21‒64†	145 (0.4)	140 (1.1)	42 (0.3)
65‒80	344 (5.9)	176 (12.1)	31 (9.0)
>81	406 (19.6)	112 (28.6)	10 (18.2)

*There was 1 death for a White patient (<1–21 year age group) and 1 death for a Hispanic patient (<1–21 year age group). †Cell percent is the case-fatality percentage for that age and race group.

**Table 3:** Characteristics and comorbidities for patients with coronavirus disease, Tennessee, USA*

Characteristic	Coronavirus disease deaths	
	Standard surveillance, n = 1,255	Chart-abstracted, n = 188
Race and ethnicity, no. (%)†		
White, non-Hispanic	787 (62.7)	109 (58.0)
Black, non-Hispanic	365 (29.1)	63 (33.6)
Hispanic (all races)	74 (5.9)	10 (5.3)
Sex, no. (%)		
F	549 (43.7)	79 (42.0)
M	706 (56.3)	109 (58.0)
Mean age, y, (SD)	73.6 (13.9)	72.7 (14.4)
Current/former smoker, no. (%)	192 (15.3)	57 (30.3)
Mean no. (SD) comorbidities	2.0 (1.5)	2.9 (1.6)
Age group, y, has any comorbidity, no. (%)†		
21–64: Nss = 284; Nca = 50	205 (72.2)	47 (94.0)
65–80: Nss = 505; Nca = 63	404 (80)	62 (98.4)
>81: Nss = 465; Nca = 74	382 (82.2)	72 (97.3)
All ages: Nss = 1,254; Nca = 188	991 (79.0)	181 (96.3)
Comorbidities, no. (%)		
Hypertension	630 (50.2)	144 (76.6)
Cardiovascular disease‡	509 (40.6)	102 (54.3)
Chronic lung disease§	257 (20.5)	56 (29.8)
Diabetes mellitus	459 (36.6)	75 (39.9)
Obesity	156 (12.4)	42 (22.3)
Cancer	54 (4.3)	20 (10.6)
Chronic renal disease	238 (19.0)	51 (27.1)
Chronic liver disease	34 (2.7)	6 (2.7)
Immunocompromised	76 (6.1)	18 (9.6)
Autoimmune diseases	21 (1.7)	8 (4.3)
HIV/AIDS	5 (0.4)	1 (0.5)

*Nca, no. patients who had chart abstraction for that age group; Nss, no. patients who had standard surveillance for that age group. †Cell percent is the percentage in that age group (i.e., for standard surveillance case-patients 21–64 years of age, the percentage is 205/284). ‡Defined as coronary artery disease, cerebrovascular, peripheral artery disease, heart failure, cardiomyopathies, valvular disease, myocarditis, and arrythmias. Hypertension is listed separately. §Defined as asthma, chronic obstructive pulmonary disease, emphysema, pulmonary hypertension, interstitial fibrosis, or sarcoidosis.

We converted comorbidities from CRFs and the chart-abstraction protocol into dichotomous variables for each condition. For the chart-abstracted deaths, we provided definitions of CVD and chronic lung disease (CLD) (Table 3) (*18**–**20*); for standard surveillance of COVID-19 deaths, we selected preexisting conditions, including CVD and CLD, on the basis of self-reports. Obesity was not included in the comorbidity analysis because the body mass index cutoffs differed between CRF and the chart-abstraction process. We calculated days to hospitalization by determining the difference in days between illness onset date and hospitalization admission date for patients who were hospitalized; we counted negative values (i.e., tested positive after hospitalization) as 0 and excluded probable cases from this calculation. We calculated days to specimen collection by determining the difference between the first specimen collection date for the PCR that had a positive result for SARS-CoV-2 and the illness onset date for confirmed case-patients; probable case-patients were excluded from this calculation.

We report patient characteristics as frequencies and proportions for categorical variables and median and interquartile range for continuous variables. We compared characteristics between groups by using χ^2^ or Fisher exact tests, as appropriate, for categorical variables and *t*-test for continuous variables and performed statistical analyses by using SAS version 9.4 (SAS Institute, https://www.sas.com).

## Results

During the study period, we identified 131,854 COVID-19 case-patients. We excluded 1,813 case-patients because of either non-Tennessee residency or death from other causes. Of the 130,040 included case-patients, 1,812 (1.4%) died from COVID-19. Deaths of COVID-19 case-patients were more likely to be in older, male, and Black case-patients than living case-patients (Table 1). The prevalence of >1 underlying condition was higher for deceased patients (64.8%) than for living patients (22.6%), and this trend was true for all age groups. There were 38,949 (30.4%) living case-patients and 369 (20.3%) deceased case-patients who did not have any comorbidity information recorded. Therefore, for the comorbidity analysis (Tables 2, 3; Appendix 2), we excluded case-patients who did not have comorbidity data (Figure 1).

**Figure 2:**
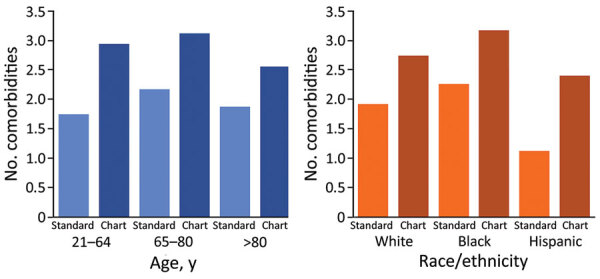
Number of comorbidities by age, race, and type of review for characteristics, comorbidities, and data gaps for coronavirus disease deaths, Tennessee, USA.

We found a difference in the case-fatality rate (CFR) for COVID-19 by race and ethnicity (White 1.9%, Black 2.6%, and Hispanic 0.5%) (Table 2). The mean age of living and deceased patients also differed by race; for deceased patients the average age was 75.6 years for White patients, 69.5 years for Black patients, and 61.3 years for Hispanic patients. After stratifying by age, we found that Black patients continued to have the highest CFR. However, Hispanic case-patients >65 years of age had a CFR similar to or higher than that for White patients (Table 2). Hispanic patients had the lowest rate of underlying medical conditions (64.3%) compared with White patients (85.7%) and Black patients (91.3%). However, Hispanic case-patients had the highest percent increase in number of comorbidities when comparing standard surveillance and chart-abstraction (Figure 2).

During March 15, 2020‒May 19, 2020, there were 355 deaths; 188 of these patients who died had complete medical records available for chart abstraction. Standard surveillance and chart abstraction had comparable frequencies of race/ethnicity, proportion of females, and age (Table 3). Chart abstraction detected a higher proportion of case-patients with >1 comorbidity (96.3%) compared with standard surveillance deaths (79.0%) (Table 3). After stratifying by race and age, we found that chart abstraction still found more comorbidities in each age group and race group (Figure 2; Appendix 2). Compared with standard surveillance, chart-abstracted deaths had a higher proportion of hypertension (76.6% vs. 50.2%), CVDs (54.3% vs. 40.6%), CLD (29.8% vs. 20.5%), cancer (10.6% vs. 4.3%), and chronic renal disease (27.1% vs. 19.0%). However, there was minimal difference in proportion of case-patients who had diabetes (39.9% vs. 36.6%). Data for chart-abstracted deaths showed a higher proportion of current/former smoking (30.3% vs. 15.3%) when compared with the standard surveillance group.

## Discussion

Our findings demonstrate that Tennessee has similar COVID-19 demographic trends to those that have been found throughout the United States (*21*,*22*). In our sample, the average age of case-patients who died was 72.9 years compared with 38.4 years for the surviving cases. Black patients were disproportionately affected by COVID-19; at the time of our analysis, 29.8% of deaths were in Black persons , but only 17.1% of the population of Tennessee identifies as Black (*23*). Hispanic patients accounted for 7.0% of deaths and 5.7% of the population in Tennessee; White patients represented 60.3% of the deaths but 73.5% of the population (*23*).

We also found major differences in CFRs for race/ethnicity: Hispanic patients had the lowest CFR (0.5%) compared with that for White patients (1.9%) and Black patients (2.6%). The lower CFR for Hispanic patients differs from US aggregate data, in which Hispanic patients have a 2.8 times higher rate of death than do White patients (*24*). In Tennessee, Hispanic patients were younger than Black and White patients, and because age is the strongest predictor of death from COVID-19 (*5*,*11*,*25*), the age difference might explain this difference in CFR for Hispanic patients in Tennessee. After stratifying by age, we found that Hispanic case-patients had CFRs similar to those for White case-patients, but Black case-patients maintained the highest CFR. In a similar fashion, CDC data have demonstrated that Hispanic patients had the largest increase in CFR once adjusted for age (*21*). Studies have demonstrated that race and ethnicity are associated with COVID-19 infection and death (*8*,*26*). However, several reviews of in-hospital death data have demonstrated that race/ethnicity is not an independent risk factor for death after admission to the hospital (*11*,*26*,*27*). Taken together, these data suggest that the disproportionate burden of COVID-19 deaths among racial and ethnic minorities is secondary to systemic health and social inequities that have limited access to chronic disease management and increased the rate of COVID infection for these populations, rather than inherent difference between races and ethnicities (*28*).

The trend in Tennessee for comorbidities for COVID-19 patients who died also mirrors the rest of the nation (*29*); 22.6% of surviving case-patients had comorbidities, compared with 64.8% of those who died. After stratifying by age, we found that a higher percentage of deceased case-patients still had an underlying condition than did living case-patients. Hypertension, CVD, CLD, cancer, chronic renal disease, diabetes, and a history of smoking were more common among deceased case-patients (Appendix 1). These correlations have been found in other studies and systemic reviews in the United States and worldwide, which have had major implications for public health messaging and vaccine allocation (*29*).

The chart review process detected higher numbers of preexisting conditions than standard surveillance. In the standard surveillance group, 79.0% had a comorbidity, compared with 96.3% in the chart-abstracted group. The difference between the standard surveillance and chart-abstracted group probably reflects issues with self-reporting and data collection. In a CDC review of COVID-19 deaths during February 12‒May 18, 2020, a total of 58.9% of patients had missing comorbidity information according to CRF-based surveillance data (*6*). In our study, 30.4% of living case-patients and 20.3% of deceased case-patients had no comorbidity data recorded. The prevalence of deceased patients who had an underlying condition in our chart-abstracted group (96.3%) is similar to that for CDC COVID-19 hospitalization records (COVID-NET), which found 405 (96.4%) of 420 deaths had an underlying medical condition (*25*). This finding emphasizes that medical chart-abstraction data collects higher rates of comorbidity data than does standard public health surveillance and is a more comprehensive representation of baseline characteristics among COVID-19 patients.

For each race and age group, we found a higher number of comorbidities recorded with chart abstraction than with standard surveillance (Figure 2). In the standard surveillance group, Hispanic patients had a lower number of comorbidities than White and Black patients. Other studies have reported mixed results; Hispanic COVID-19 patients who died had more or fewer comorbidities than non-Hispanic patients (*25*,*27*). In our chart-abstracted group, the total number of comorbidities for Hispanic patients was twice that of the standard surveillance group, which was the largest increase for race/ethnicity (Appendix 2). For the standard surveillance group, information was gathered by in person or telephone conversations. Therefore, language barriers and concerns about disclosure of information are 2 possible explanations for the lower number of comorbidities recorded. It has been shown that non-English‒speaking patients are more likely to have inaccurate medical information, to receive lower quality care, and are at a higher risk for medical errors that result in harm (*30*,*31*). Taken together, our findings demonstrate the value of chart abstractions to obtain accurate information for Hispanic and non‒English speaking patients during disease surveillance.

We observed notable trends in the prevalence of certain comorbidities in the standard surveillance deaths compared with the chart-abstracted deaths. For example, hypertension, CVD, and CLD were detected in higher frequencies in the chart-abstracted group, and diabetes had similar rates in chart-abstraction and standard surveillance. Similarly, in multiple studies worldwide comparing self-reports and medical records, diabetes was the disease with the highest concordance (*32*–*34*); hypertension and CVD are frequently underreported in self-reports (*32*,*35*). Therefore, diabetes is probably better captured by standard interview-based surveillance than other comorbidities. A meta-analysis of 87 studies determined that diabetes was the comorbidity that had the highest association with COVID-19 deaths (*36*). Diabetes certainly increases risk for COVID-19 deaths, but it is possible that the high accuracy of diabetes disease reporting could disproportionately increase the association between diabetes and COVID-19 death compared with other comorbidities. In addition, there is mixed evidence about whether hypertension is an independent risk factor for death (*29*); part of this difference could be explained by data collection and inaccurate reporting by patients.

One limitation of our study is that we used a convenience sample of COVID-19 cases collected by the TDH, which led to collection biases. For our comorbidity analysis, we excluded cases without any comorbidity information, which led to selection bias. Our chart-abstracted study occurred at the beginning of the pandemic and does not capture the burden of COVID-19 for certain ethnic and geographic groups who had more cases later in the pandemic. For example, there were 126 deaths in Hispanic COVID-19 patients and only 10 patients in the Hispanic chart-abstracted group. Furthermore, our chart-abstracted study relied on medical charts, which created selection bias and missed patients who died outside hospitals. There might be certain groups who are more likely to have out-of-hospital deaths, but these deaths were not evaluated in our study. The data collection process was different for the standard surveillance deaths and the chart-abstracted deaths, which limited the validity of comparing the frequencies of characteristics and comorbidities. Despite these limitations, we analyzed a large number of patients, and analyzed COVID-19 demographic trends for Tennessee for comparison to other states. In addition, our chart-abstraction analysis is a description of a public health study that met its goal to capture additional information compared with standard surveillance.

Our chart-based analysis showed that comorbidities related to COVID-19 deaths are more prevalent than those identified by standard public health disease surveillance. Furthermore, certain patient information tends to be reported less accurately in standard surveillance than in chart-based analysis. However, chart-based reviews are labor and time intensive, and the COVID-19 pandemic has highlighted how public health agencies are understaffed and underfunded (*9*). One solution for the challenges of data collection in public health disease surveillance is expansion of electronic case reporting. This type of reporting uses an interoperable, shared service infrastructure to enable automated real-time exchange of information from electronic medical records to public health agencies (*37*). In traditional case reporting, the most cases are reported from laboratories who lack detailed information on case demographics and often send their reports by paper copy. Therefore, public health departments receive incomplete information, which creates data gaps and distorted data, which is also apparent in our findings. In contrast, electronic case reporting provides faster and more complete data from healthcare institutions while decreasing the burden on reporters and public health departments (*37*). During the COVID-19 pandemic, there has been increased uptake in electronic case reporting, and continuing this trend is essential for effective disease surveillance (*38*).

Throughout the COVID-19 pandemic, data collected by public health agencies have been integral in identifying trends and providing information to health agencies (*39*). The surveillance data from Tennessee demonstrate trends in age, comorbidities, and race/ethnicity that mirror the rest of the country, and this data been used to protect those at highest risk for severe COVID-19 disease. Our study showed that chart abstraction collects more comorbidity data than standard public health disease surveillance. In addition, certain diseases and patient groups are frequently underreported in standard surveillance, which skews public health data. These data gaps can miss at risk groups and can lead to unadvised public health action. Investment in data collection infrastructure that collects more timely and complete data will equip public health institutions, governmental organizations, and the scientific community with accurate information required to mitigate disease burden in COVID-19 and future outbreaks.

Appendix 1 Case report form for preexisting conditions and additional comorbidity items from chart review for characteristics, comorbidities, and data gaps for coronavirus disease deaths, Tennessee, USA.

Appendix 2 Additional information on characteristics, comorbidities, and data gaps for coronavirus disease deaths, Tennessee, USA.
