# Supplementary material for: Characteristics, Comorbidities, and Data Gaps for Coronavirus Disease Deaths, Tennessee, USA
Source: Emerg Infect Dis. 2021 Oct;27(10):2521–8. doi: 10.3201/eid2710.211070 (PMC8462317; doi:10.3201/eid2710.211070)
Supplement: Appendix 1 — Case report form for preexisting conditions and additional comorbidity items from chart review for characteristics, comorbidities, and data gaps for coronavirus disease deaths, Tennessee, USA. [file 21-1070-Techapp-s1.pdf]

Article DOI: https://doi.org/10.3201/eid2710.211070

Characteristics, Comorbidities, and Data Gaps for Coronavirus Disease Deaths, Tennessee, USA

Appendix 1

Case Report Form, Preexisting Conditions Section

This form was completed in standard surveillance and chart-abstraction

**Did they have any underlying medical conditions and/or risk behaviors?** ☐Yes ☐No ☐ Unknown				
Diabetes Mellitus	☐Yes ☐No ☐Unk	Immunosuppressive condition	☐Yes ☐No ☐Unk
Hypertension	☐Yes ☐No ☐Unk	Autoimmune condition	☐Yes ☐No ☐Unk
Severe obesity (BMI ≥40)	☐Yes ☐No ☐Unk	Current smoker	☐Yes ☐No ☐Unk
Cardiovascular disease	☐Yes ☐No ☐Unk	Former smoker	☐Yes ☐No ☐Unk
Chronic Renal disease	☐Yes ☐No ☐Unk	Substance abuse or misuse	☐Yes ☐No ☐Unk
Chronic Liver disease	☐Yes ☐No ☐Unk	Disability (neurologic, neurodevelopmental, intellectual, physical, vision or hearing impairment) If yes, specify: ______________	☐Yes ☐No ☐Unk
Chronic Lung disease (asthma/emphysema/COPD)	☐Yes ☐No ☐Unk		
Other chronic diseases If yes, specify: ______________	☐Yes ☐No ☐Unk		
Other underlying condition or risk behavior, specify: ______________	☐Yes ☐No ☐Unk	Psychological/psychiatric condition If yes, specify: ______________	☐Yes ☐No ☐Unk

Supplemental Chart Review: Additional comorbidity questions

Enhanced Mortality Surveillance

Collapse Subsections

Note

Central Office is completing Enhanced Mortality Surveillance to capture additional information about deceased patients.
This section should only be completed if the patient is deceased due to this illness.

Comorbidities

Current carcinoma (other than skin):
If carcinoma, specify:
End Stage Liver Disease (e.g., cirrhosis):
End Stage Renal Disease (ESRD):
Coronary Artery Disease:
Other Heart disease (e.g., cardiomyopathy, arrhythmia, etc.):
HIV/AIDS:
Asthma:
Chronic obstructive pulmonary disease (COPD):
Other chronic lung disease (e.g, idiopathic pulmonary fibrosis, sarcoidosis):
If other chronic lung disease, specify:
Obstructive Sleep Apnea (OSA):
Organ or bone marrow transplant (history):
Neuromuscular/neurodegenerative disease:
Peripheral Vascular Disease:
Cerebrovascular Disease:
Other comorbidity(ies):
Was the patient DNR?:

Exposures

Current chronic oral steroids (e.g., Prednisone = 10mg/day):
Current Chemotherapy:
If yes, specify therapy:
Current immunosuppressive treatment other than steroids:
If yes, specify therapy:
Dialysis:
