# Supplementary material for: Characteristics, Comorbidities, and Data Gaps for Coronavirus Disease Deaths, Tennessee, USA
Source: Emerg Infect Dis. 2021 Oct;27(10):2521–8. doi: 10.3201/eid2710.211070 (PMC8462317; doi:10.3201/eid2710.211070)
Supplement: Appendix 2 — Additional information on characteristics, comorbidities, and data gaps for coronavirus disease deaths, Tennessee, USA. [file 21-1070-Techapp-s2.pdf]

Article DOI: https://doi.org/10.3201/eid2710.211070

Characteristics, Comorbidities, and Data Gaps for Coronavirus Disease Deaths, Tennessee, USA

Appendix 2

Table 1. Characteristics and comorbidities of living and deceased patients who had coronavirus disease and completed comorbidity information

Characteristic	Outcome: Living, n = 89,279	Outcome: Deceased, n = 1,443
Race and ethnicity, no. (%)*		
White, non-Hispanic	46,677 (52.3)	896 (62.1)
Black, non-Hispanic	16,699 (18.7)	428 (29.7)
Hispanic (all races)	17,084 (19.1)	84 (5.8)
Sex, no. (%)		
F	45,857 (51.4)	628 (43.5)
M	42,950 (48.1)	815 (56.5)
Unknown	472 (0.5)	0 (0.0)
Age, y (mean)	38.9 (19.3)	73.5 (13.9)
Current/former smoker, no. (%)	10,731 (12.0)	249 (17.3)
Mean (SD) no. comorbidities	0.5 (0.9)	2.1 (1.6)
Age group, y, has any comorbidity, no. (%)	28,898 (32.4)	1,172 (81.2)
<1–20 (living =15,478, deceased = 2)	1,585 (10.2)	1 (50.0)
21–64 (living = 63,981, deceased = 334)	20,832 (32.6)	252 (75.4)
65–80 (living = 7,657, deceased = 568)	5,039 (65.8)	466 (82.0)
≥81 (living = 2,122, deceased = 539)	1,437 (67.7)	453 (84.0)
Pending (living = 41, deceased 0)	5 (12.2)	Not available
Comorbidities, no. (%)		
Hypertension	11,675 (12.1)	774 (53.6)
Cardiovascular disease†	4,672 (5.2)	611 (42.3)
Chronic lung disease‡	5,935 (6.7)	313 (21.7)
Diabetes mellitus	7,393 (8.3)	534 (37.0)
Obesity	2,899 (3.3)	198 (13.7)
Cancer	330 (0.4)	74 (5.1)
Chronic kidney disease	1,112 (1.3)	289 (2.0)
Chronic liver disease	614 (0.7)	39 (2.7)
Immunocompromised	1,309 (1.5)	94 (6.5)
Autoimmune diseases	1,089 (1.2)	29 (2.0)
HIV/AIDS	21 (0.02)	6 (0.4)

*Race and ethnicity breakdown for other races is the remaining group breakdown not listed.
†Defined as coronary artery disease, cerebrovascular, peripheral artery disease, heart failure, cardiomyopathies, valvular disease, myocarditis, and arrythmias. Hypertension is listed separately.
‡Defined as asthma, chronic obstructive pulmonary disease, emphysema, pulmonary hypertension, interstitial fibrosis, or sarcoidosis.

Table 2. Breakdown of race-stratified comorbidities among living and deceased coronavirus disease patients who had comorbidities and ethnicity information*

	Living, n = 80,460			Deceased, n = 1,408		
Characteristic	White, non-Hispanic, n = 46,677	Black, non-Hispanic, n = 16,669	Hispanic, n = 17,084	White, non-Hispanic, n = 896	Black, non-Hispanic, n = 428	Hispanic, n= 84
Median age, y, (interquartile range)	40 (25–57)	37 (25–52)	32 (21–45)	78 (69–86)	71 (61–80)	63 (51–73)
Mean (SD) days to hospitalization	4.2 (5.6)	3.7 (5.3)	4.4 (4.9)	3.6 (5.8)	3.2 (6.5)	5.2 (4.9)
Mean (SD) days to specimen collection	2.3 (3.8)	2.3 (4.2)	2.8 (4.7)	2.1 (4.6)	2.3 (4.3)	3.2 (3.9)
Age group, y, median (interquartile range)						
21–64	0 (0–1)	0 (0–1)	0 (0–0)	2 (0–3)	2 (1–3)	1 (0–2)
65–80	1 (0–2)	1 (0–2)	1(0–1)	2 (1–3)	3 (1–4)	1 (0–2)
≥81	1 (0–2)	2 (1–3)	1 (0–2)	2(1–3)	2 (1–3)	1 (0–2)
All ages	0 (0–1)	0 (0–1)	0 (0–0)	2 (1–3)	2 (1–3)	1 (0–2)
Comorbidities, no.(%)						
Current/former smoker	6,543 (14.0)	2,076 (12.4)	1,458 (8.5)	162 (18.1)	71 (16.6)	8 (9.5)
Hypertension	6,908 (14.8)	3.268 (19.6)	904 (5.3)	454 (50.7)	280 (65.4)	27 (32.1)
Cardiovascular disease	3,197 (6.9)	974 (5.8)	238 (1.4)	404 (45.1)	182 (42.5)	14 (16.7)
Chronic lung disease	3,676 (7.9)	1,394 (8.4)	511 (3.0)	217 (24.2)	77 (18.0)	11 (13.1)
Diabetes mellitus	4,036 (8.7)	1,959 (11.7)	949 (5.6)	289 (32.3)	200 (46.7))	34 (40.5)
Cancer	239 (0.5)	64 (0.4)	16 (0.1)	34 (3.7)	36 (8.4)	2 (2.4)
Chronic renal disease	695 (1.5)	300 (1.8)	66(0.4)	151 (16.9)	124 (28.9)	7 (8.3)
Chronic liver disease	422 (0.9)	94 (0.6)	67 (0.4)	20 (2.2)	14 (3.3)	2 (2.4)
Immunocompromised	851 (1.8)	301 (1.8)	79(0.5)	56 (6.3)	31 (7.3)	2 (2.4)
Autoimmune diseases	841 (1.8)	144 (0.9)	44 (0.3)	23 (2.6)	5 (1.2)	0 (0)

Table 3. Breakdown of race-stratified comorbidities for and deceased coronavirus disease patients who had comorbidities and ethnicity information

	White, non-Hispanic, n = 896		Black, non-Hispanic, n = 428		Hispanic, all races, n = 84	
Characteristic	Standard, n = 787	Chart, n =109	Standard, n = 365	Chart, n = 63	Standard, n = 74	Chart, n = 10
Age, mean (SD)	76.6 (12.1)	75.8 (12.5)	69.6 (14.8)	69.4 (14.2)	61.3 (16.0)	60.8 (24.1)
Mean no. comorbidities (SD)	1.9 (1.5)	2.7 (1.4)	2.2 (1.5)	3.1 (1.7)	1.2 (1.3)	2.4 (1.4)
Mean no. days to hospitalization (SD)	2.6 (5.3)	2.7 (4.4)	2.7 (6.4)	2.5 (4.2)	4.2 (4.8)	4.2 (5.0)
Mean (SD) days to specimen collection	2.0 (4.6)	2.5 (4.1)	2.2 (4.3)	2.5 (4.0)	3.2 (3.9)	3.5 (4.4)
Current/former smoker, no. (%)	127 (16.1)	35 (32.1)	52(14.3)	19 (30.2)	7 (9.5)	1 (10.0)
Comorbidities, no. (%)						
Hypertension	371 (47.2)	83 (76.2)	227 (62.2)	53 (84.1)	21 (28.4)	6 (60.0)
Cardiovascular disease	343 (43.6)	6 (56.0)	148 (40.6)	34 (55.0)	9 (12.2)	5 (50.0)
Chronic lung disease	187 (23.8)	30 (27.5)	56 (15.3)	21(33.3)	8 (10.8)	3 (30.0)
Diabetes mellitus	255 (32.4)	34 (31.2)	167 (45.8)	33 (52.4)	28 (37.8)	6 (60.0)
Cancer	20 (2.5)	13 (11.9)	30 (8.2)	6 (9.5)	2 (2.7)	0 (0)
Chronic renal disease	125 (15.9)	26 (23.9)	102 (28.0)	22 (34.9)	5 (6.8)	2 (20.0)
Chronic liver disease	17 (2.2)	3 (2.8)	12 (3.3)	2 (3.2)	2 (2.7)	0 (0)
Immunocompromised	47 (6.0)	9 (8.3)	24 (6.6)	7 (11.1)	1 (1.4)	1 (10.0)
Autoimmune diseases	19 (2.4)	4 (3.7)	2 (0.6)	3 (4.8)	0	0

Table 4. Breakdown of age-stratified comorbidities for deceased coronavirus disease 19 patients who had comorbidities and ethnicity information

Characteristic	Standard, n = 284	Chart, n =50	Standard, n = 505	Chart, n = 63	Standard, n = 545	Chart, n = 74
Mean no. comorbidities (SD)	1.7 (1.5)	2.9 (1.8)	2.2 (1.6)	3.2 (1.7)	1.9 (1.4)	2.5 (1.2)
Mean no. days to hospitalization (SD)	3.3 (5.2)	3.4 (4.9)	3.3 (6.7)	3.3 (4.5)	1.8 (4.2)	1.7 (3.6)
Mean no. days to specimen collection (SD)	2.8 (4.6)	3.0 (4.3)	2.2 (4.2)	2.9 (4.3)	1.6 (4.5)	2.0 (3.8)
Current/former smoker, no. (%)	37 (13.0)	22 (44.0)	112 (22.2)	21 (33.3)	43 (9.3)	13 (18.9)
Obesity, no. (%)	72 (25.3)	19 (38.0)	62 (12.3)	16 (25.4)	22 (4.7)	7 (9.5)
Comorbidities, no. (%)						
Hypertension	132 (46.5)	35 (70.0)	246 (48.7)	49 (77.8)	252 (54.2)	60 (81.1)
Cardiovascular disease	65 (22.9)	18 (36)	224 (44.4)	40 (63.5)	220 (47.3)	43 (58.1)
Chronic lung disease	47 (16.6)	16 (32.0)	116 (23.0)	22 (34.9)	94 (20.2)	17 (23.0)
Diabetes mellitus	113 (39.8)	28 (56.0)	208 (41.2)	32 (50.8)	138 (29.7)	15 (20.3)
Cancer	12 (4.2)	6 (12.0)	27 (5.4)	7 (11.1)	15 (3.2)	7 (9.5)
Chronic renal disease	41 (14.4)	11 (22.0)	104 (20.6)	17 (27.0)	93 (20.0)	23 (31.1)
Chronic liver disease	12 (4.2)	2 (4.0)	17 (3.4)	3 (4.8)	5 (1.1)	0 (0)
Immunocompromised	28 (9.9)	6 (12.0)	27 (5.4)	8 (12.7)	21 (4.5)	4 (5.4)
Autoimmune diseases	5 (1.8(	2 (4.0)	13 (2.6)	3 (4.8)	3 (0.7)	3 (4.1)
